# Clinical Evaluation of self and professionally applied desensitizing agents in relieving dentin hypersensitivity after a single topical application: A Randomized Controlled Trial

**DOI:** 10.4317/jced.51439

**Published:** 2014-10-01

**Authors:** Srinivasan-Raj Samuel, Sachin G. Khatri, Shashidhar Acharya

**Affiliations:** 1 Senior Lecturer, Department of Public Health Dentistry, Thai Moogambigai Dental College Chennai, Tamil Nadu, India; 2 Professor and Head. Department of Public Health dentistry, Manipal College of Dental Sciences, Manipal, Karnataka, India

## Abstract

Objectives: The objective of this study is to evaluate the efficacy of self and professionally applied desensitizing agents in relieving dentinal hypersensitivity after single direct topical application. Study Design: This was a randomized controlled trial conducted among 57 patients. 8% Arginine paste was self-applied by the subject and Gluma desensitizer was applied by investigator. Numeric rating scale was used to measure hypersensitivity after tactile stimulus, Schiff scale was used for cold and air blast stimuli respectively. Scores were recorded at baseline, immediately, 15 and 30 days after the application. Friedman, Wilcoxon test as post hoc was used to analyze within group differences, between group differences analyzed using Mann Whitney U test (P<0.05 considered significant). Results: 8% Arginine paste elicited significantly higher reductions in sensitivity (P<0.05) than that of Gluma group at all follow ups. There was a significant decrease in hypersensitivity for both the groups from baseline till final follow-up (P<0.05) for all three stimuli. 8% Arginine paste was found to be more effective than Gluma desensitizer in providing immediate relief from dentine hypersensitivity and also sustained the effect significantly for a period of 30 days. Conclusions: Self applied 8% Arginine paste is effective than professionally applied Gluma desensitizer in relieving dentinal hypersensitivity immediately and over a period of one month.

** Key words:**Dentine hypersensitivity, arginine, gluma, desensitizing agents.

## Introduction

Dentinal hypersensitivity [DH] is a common condition in daily practice, especially in patients who have abrasion, attrition, gingival recession and erosion of teeth. It usually affects individuals in their thirties, although it can affect individuals in the age range of 20-50 years (1). The buccal-cervical regions of the canine and premolar teeth, also sites which are most susceptible to gingival recession are the ones most commonly affected by dentinal hypersensitivity (2). The discomfort experienced by individuals suffering from DH is short and sharp pain caused by exposed dentinal tubules in response to stimuli like thermal, tactile, osmotic, chemical or evaporative; that cannot be ascribed to any disease. Several theories like transduction, neural stimulation and hydrodynamic theories have been proposed for the causal of DH, but the most widely accepted is the hydrodynamic theory (3). Customarily, the first treatment option for DH would be recommending desensitizing toothpaste. When used regularly over a few weeks many individuals feel relieved, although it is not a permanent solution. If the self-use prescriptions fail to work, professional methods of sealing the dentinal tubules would be considered. Self-applied desensitizing agents have the advantage of immediate availability for treatment as compared to those which are applied by a professional. Nevertheless, professionally applied desensitizing agents theoretically have the advantage of immediate relief from the symptoms. The disadvantage with the self-applied agents is the time required for the relief of symptoms associated with DH. It usually takes about 2-4 weeks for remission of symptoms (4). Professionally sealing the exposed dentinal tubules is restricted to in-office products like, HEMA-G [Hydroxyethyl Methacrylate and Glutaraldehyde], fluoride varnish, fluoride iontophoresis and lasers. These materials have been proved to provide relief from DH in one or more professional applications. HEMA-G prevents DH by coagulating the proteins and amino acids within the dentinal tubules (5). Nevertheless, the number of people seeking professional help is low because of the ever increasing cost of visiting a dentist for treatment. Two major approaches are presently available in treating DH. First is to interrupt the neural response to triggering factors; secondly, by occluding the exposed dentinal tubules thereby blocking the hydrodynamic mechanism (6). Markowitz and Pashley suggested that the treatment should focus on increasing the surface mineral density of exposed dentin, use of calcium rich material for sealing exposed dentin, accelerate natural desensitizing process of occluding open tubules (7). The recently introduced Pro-arginine technology for the treatment of DH has been found to be effective in reducing the DH, either self-applied or professionally applied. The mechanism of action of Pro-arginine is that it mimics natural desensitizing process leading to spontaneous occlusion of open dentinal tubules by the formation of calcium and phosphate plugs (8). This paste has been introduced in a concentration of 8.0% for relieving DH by both self and professional application. Thus the purpose of this double blind, randomized clinical trial was to evaluate the clinical efficacy of self-applied Colgate sensitive Pro-Relief [Colgate-Palmolive, Guildford, Surrey, UK], and professionally applied Gluma Desensitizer [Heraeus Kulzer, Armonk, NY, USA] in relieving the dentinal hypersensitivity after a single direct topical application over a period of one month.

## Material and Methods

This study was a randomized, double blind, single site controlled clinical trial conducted in the Comprehensive Dental Care Center, Udupi; Karnataka from July 2012 till March 2013. Institutional review board of Kasturba Hospital Ethics Committee reviewed, approved the study protocol [IEC 161/ 2012]. Individual tooth was the unit of the study in this trial. 57 patients [114 teeth] including 32 men and 25 women between the age group of 20 and 50 years [Mean age 33.9 ± 7.8] were recruited. Principal investigator clinically evaluated all participants to confirm that they had DH and included eligible subjects in the trial based on the following inclusion and exclusion criteria. Inclusion criteria were as follows: subjects between the age group of 20-50 years, presenting with complaint of hypersensitivity, those with cervical erosion/abrasion or gingival recession and those having a minimum of two teeth with dentinal hypersensitivity. Subjects were excluded from the study if they had any history of allergies or idiosyncrasies to dentifrice ingredients, gross oral pathology, chronic diseases, advanced periodontal disease, treatment for periodontal disease [within previous 12 months], hypersensitive teeth with mobility greater than one [Miller, 1938] and those with teeth that have extensive/defective restorations [including prosthetic crowns], suspected pulpitis, caries, cracked enamel, or those teeth being used as abutments for removable partial dentures and who are current users of anticonvulsants, antihistamines, antidepressants, sedatives, tranquilizers, anti-inflammatory drugs, or daily analgesics. Informed consent was obtained before the commencement of the trial. Subjects not willing to provide consent were not included in the trial.

The sample size was determined based on the pilot study conducted among 10 participants [20 teeth]. 10 teeth each were randomly allotted to both Gluma and Arginine group. The pooled variance was calculated based on the results obtained. A difference of 1 in the Schiff scores between the baseline and the follow up after 30 days in the Schiff sensitivity scale (9) was considered to be indicative of clinically accepted success. Final estimated sample was 46 per group.

Randomization: A total of 114 teeth were included in the trial and were randomly allocated to the two groups; (54) Gluma and (60) Arginine respectively using fish bowl randomization. Recording of DH at baseline, immediately, 15 days and after 30 days were recorded by second investigator who was blinded as to which group the particular tooth belonged. Each tooth was evaluated for tactile, air blast and cold stimuli on causing dentinal hypersensitivity. A trained recorder helped the investigator in recording the DH scores at each follow up. All the subjects were instructed to use non fluoridated toothpaste, as it can confound the results. This was ensured by prescribing non-fluoridated toothpaste [Miswak] to be purchased from the pharmacy. Subjects were also instructed not to use any mouthwash during the course of the study.

Assessment of tactile, air blast and cold hypersensitivity: Tactile stimuli were evaluated by running an explorer [17/23] perpendicular to buccal-cervical/ exposed root surface. The score was recorded on a numerical rating scale ranging from 1-10 [0 no pain, 1-3 mild, 4-6 moderate, 7-10 severe]. Air was delivered from a standard dental unit air syringe at 40 psi [± 5 psi] and 70°F [± 3°F] directed at the exposed buccal surface of the hyper-sensitive tooth for two seconds from a distance of approximately 10 mm to elicit air blast hypersensitivity. Cold sensitivity was assessed by injecting 0.2 ml of ice cold water from a pre cooled syringe onto the tooth which is isolated using cotton rolls. The Schiff Cold Air Sensitivity Scale (9) was used to assess subject response to air blast and cold stimulus. Evaluation for various stimuli was performed with a time interval of five minutes bet-ween each. Subjects who gave a minimum score of 3 for numeric rating scale [tactile] and a minimum of 2 in Schiff’s sensitivity scale [air blast/ cold stimuli] during the baseline examination were recruited in the study. The tooth to be treated were isolated from the adjacent teeth using cotton rolls and 8% Arginine paste was applied by taking small increment of the paste in a finger and massaged over the buccal-cervical area of the tooth by the patient for a minute after which the patient is asked to rinse gently with water. Gluma desensitizer was applied using an applicator tip onto the tooth being treated by the investigator and allowed to dry for a minute after which the patient was asked to rinse gently with water. Subjects were recalled and dentinal hypersensitivity was evaluated for tactile, air blast and cold stimuli immediately, 15, 30 days after the application of desensitizing agents. All the patients were communicated on their cell phone on day before the follow up to remind them, further a short message service [SMS] was sent on the morning of the follow up to avoid loss as much as possible. This method was very effective in motivating the patients.

Statistical methods: Normality of the data was analyzed for each group using Shapiro Wilk test [*p*<0.001]. Data was found to be non-normally distributed, hence non parametric test were employed using SPSS 16.0 for windows [SPSS Inc, Chicago, IL, USA] to analyze them. The within group differences at different time frames were compared using Friedman test and Wilcoxon signed rank test with bonferroni correction used as *post hoc*. Comparison of the between groups based on baseline assessments and subsequent follow ups were performed using Mann-Whitney U test to assess the change in mean score from baseline. All the statistical tests were two sided and P value was adjusted [Bonferroni method]; α = 0.05 was used as an overall experiment error rate.

## Results

57 subjects with a mean age of 33.9 ±7.8 years were recruited in the study and five [9%] were lost to follow up [three subjects from Gluma group, two from Arginine], thus were excluded from the final analysis. Finally, 52 subjects [104 teeth] completed the 30 days clinical trial. Distribution of study population according to age and gender of subjects in all the three groups is presented in  Table 1. Friedman test showed a statistically significant reduction [*P*<0.05] in mean score for DH from baseline to all subsequent follow ups within both treatment group. Wilcoxon test with bonferroni correction used as a post hoc analysis showed a significant reduction [*P*<0.0167] in the mean score for DH from baseline-immediately, baseline-15 days and baseline-30 days for both Arginine and Gluma groups ( Table 2). Between groups comparison for Gluma and Arginine is presented in  Table 3. Mann-Whitney U test found significant reductions [*P*<0.05] in the mean DH among the two groups. When the DH was elicited by all three stimuli, Arginine group significantly reduced [*P*<0.05] DH during all the follow ups as compared to that of Gluma group, except for air blast sensitivity at 15 days follow up.

**Table 1:**
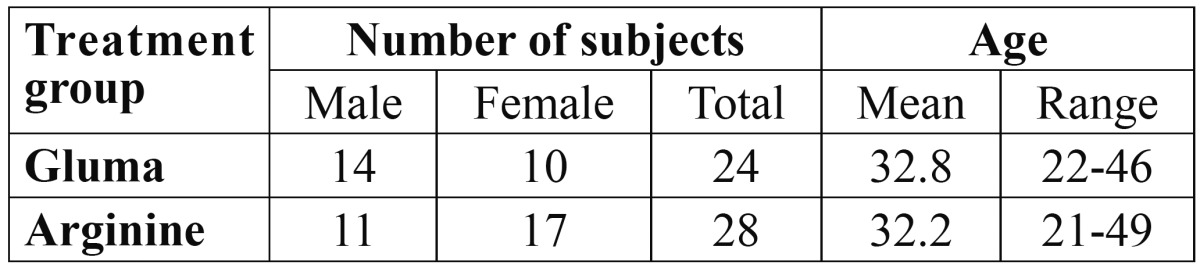
Summary of age and gender of all the subjects who completed the trial.

**Table 2:**
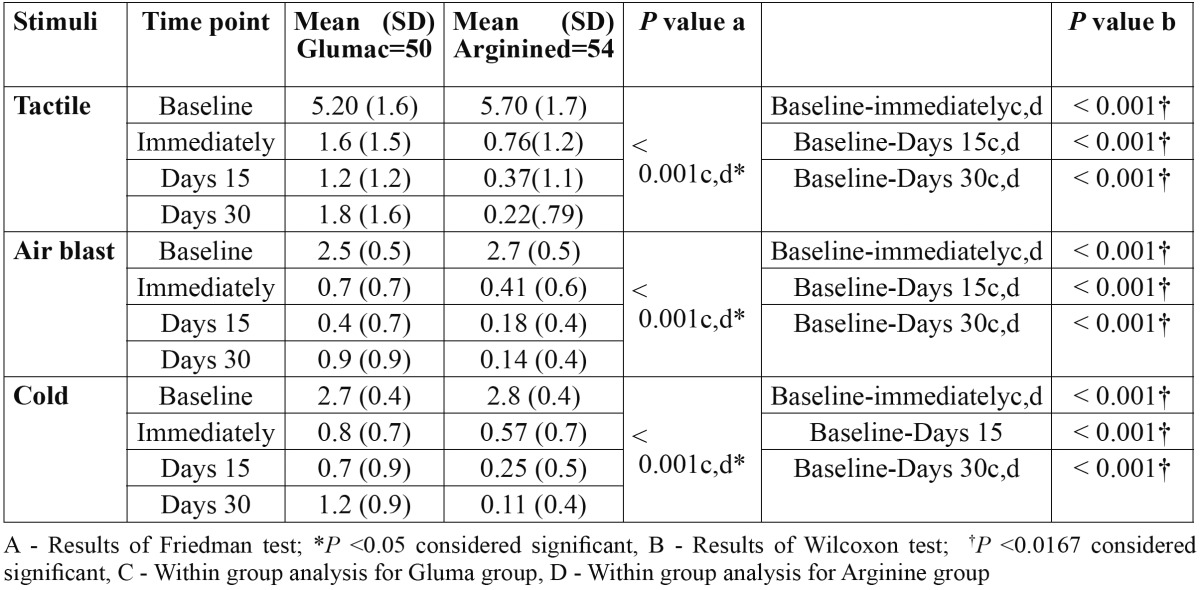
Within group comparisons of dentinal hypersensitivity scores (Mean ± SD) to various stimuli at three different intervals in Gluma and Arginine group.

**Table 3:**
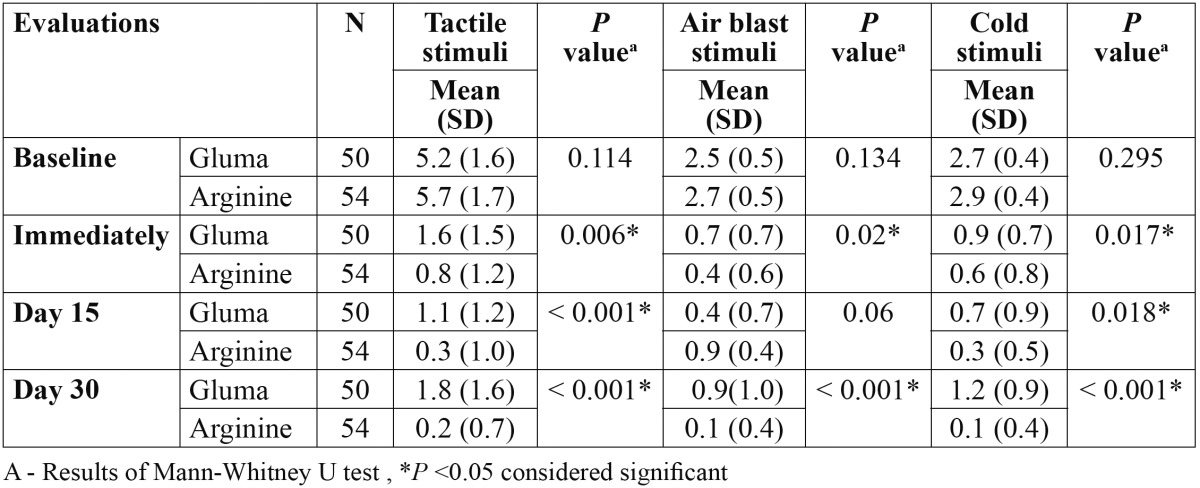
Comparison of dentinal hypersensitivity scores (Mean ± SD) for tactile, airblast and cold stimuli at three different intervals among Gluma and Arginine groups.

## Discussion

In this study the efficacy of two desensitizing agents were compared in a single site, controlled, double blind, and parallel design trial to reduce DH after single direct self and professionally applied agents. The two desensitizing agents used are completely different in their chemical composition and mode of action. The professionally applied agent, Gluma desensitizer a proven desensitizing agent, acts by coagulating the proteins within the dentinal tubule (5,10). The self-applied agent, Colgate sensitive pro relief contains a novel desensitizing agent, 8% arginine, bicarbonate and calcium carbonate, thereby, forming dentin plugs to prevent DH.

The results of the present study demonstrate comparable clinical effectiveness among both the desensitizing agents with significant reductions in the measure of dentine hypersensitivity till the 30th day after single direct topical application. Prominent and clinically relevant reductions [*p*<0.05] in the pain caused by tactile, air-blast and cold stimuli were found in both the groups. However, when compared between the groups, Arginine contai-ning paste was found to be more effective in reducing DH. Arginine paste was also found to sustain the immediate desensitization achieved to period of 30 days as compared to Gluma. This result is in accordance with results from studies, which the desensitization achieved through the use of Pro-arginine technology is immediate and lasts up to 28 days (11,12). The possible explanation for the result observed in the present study may relate to the effectiveness of arginine in sealing the dentinal tubules, its acid resistant nature and the depth of the plug [2µm] formed within the tubule as found in various confocal and scanning electron microscopic studies (13). Result of similar research evaluating the efficacy of Arginine in providing instant relief after single direct topical self-application is in concordance with the results obtained in this study (14,15). In a study by Olusile, Gluma was found to be most effective in the first 24 hour period (16). Ozen T and others evaluated the efficacy of Gluma, Ultra EZ and Duraphat in reducing dentinal hypersensitivity in short term treatment period against a placebo. They found that Gluma performed best, but without any significant difference with others (17). Although, Gluma is considered to be an effective in-office desensitizing agent, it was not found to be as effective as Colgate sensitive pro relief in our study. One of the reasons probably could be the lack of acid resistance of Gluma which can explain the lack of longevity observed. The cervical areas of the tooth are the ones most susceptible to DH, but are also known for plaque stagnation; hence, Gluma could have been lost because of the acids produced from the plaque accumulated.

DH is a common disease, whose prevalence and symptoms has been studied in a variety of population across the globe. There are no established parameters to measure DH, and reporting of DH is also not homogeneous among investigators. Millions of people are affected by DH and for some it also affects the QoL and daily activities (18). The prevalence of DH is expected to increase, so is the demand for effective management of DH. Medicine and dentistry has evolved into a new era of prolonging life expectancy and preserving life. The longer the person lives and retains teeth in older age, more is the chance for DH. Reasons for the increase in prevalence may also be attributed to the lifestyle alterations, habits and behavioral changes. It is quite striking that despite the extended prevalence, not many opt for clinical management or use of desensitizing agents (19). The rationale behind the reduced use of desensitizing agents may be because of the high cost involved in visiting a dentist, costlier desensitizing agents and also because of objectionable taste and stinging effect on oral mucosa, which discourages tolerability and continuous use.

To the best of our knowledge this is the first study to evaluate the efficacy of self-application and professional application in reducing DH after single direct topical application using arginine containing tooth paste and Gluma desensitizer. Negative control could not be used in our study, because it was not ethically and morally right to deny treatment as DH affects the daily activities, but it’s the major limitation of this study. Lack of control does affect the interpretation of the results of this study with respect to the response measured. Thus, authors recommend clinical trials testing the efficacy of desensitizing agents to have negative control arm to further strengthen the study findings. The main challenge for epidemiological comparisons is the definition and the clinical methodology employed, each study varies from investigator to investigator, no gold standard exists as to carry out desensitizing studies.

## Conclusions

In conclusion, this study demonstrates the efficacy of self-applied Arginine containing toothpaste [Colgate sensitive pro relief] in reducing the DH and sustaining it reasonably for a period of 30 days when compared to professionally applied Gluma desensitizer after a single topical application. It can serve as an effective agent in treating DH rather than vising the dentist for the treatment of the same, thereby, also reducing the costs involved in visiting a dentist.
